# Modulation of Calcium-Dependent Inactivation of L-Type Ca^2+^ Channels via β-Adrenergic Signaling in Thalamocortical Relay Neurons

**DOI:** 10.1371/journal.pone.0027474

**Published:** 2011-12-02

**Authors:** Vladan Rankovic, Peter Landgraf, Tatyana Kanyshkova, Petra Ehling, Sven G. Meuth, Michael R. Kreutz, Thomas Budde, Thomas Munsch

**Affiliations:** 1 Institute of Physiology, Otto-von-Guericke University Magdeburg, Germany; 2 PG Neuroplasticity, Leibniz Institute for Neurobiology, Magdeburg, Germany; 3 Institute of Physiology I, Muenster, Germany; 4 Institute of Neuropathophysiology and Neurology Clinic – Department of Inflammatory Disorders of the Nervous System and Neurooncology, Muenster, Germany; Julius-Maximilians-Universität Würzburg, Germany

## Abstract

Neuronal high-voltage-activated (HVA) Ca^2+^ channels are rapidly inactivated by a mechanism that is termed Ca^2+^-dependent inactivation (CDI). In this study we have shown that β-adrenergic receptor (βAR) stimulation inhibits CDI in rat thalamocortical (TC) relay neurons. This effect can be blocked by inhibition of cAMP-dependent protein kinase (PKA) with a cell-permeable inhibitor (myristoylated protein kinase inhibitor-(14–22)-amide) or A-kinase anchor protein (AKAP) St-Ht31 inhibitory peptide, suggesting a critical role of these molecules downstream of the receptor. Moreover, inhibition of protein phosphatases (PP) with okadaic acid revealed the involvement of phosphorylation events in modulation of CDI after βAR stimulation. Double fluorescence immunocytochemistry and pull down experiments further support the idea that modulation of CDI in TC neurons via βAR stimulation requires a protein complex consisting of Ca_V_1.2, PKA and proteins from the AKAP family. All together our data suggest that AKAPs mediate targeting of PKA to L-type Ca^2+^ channels allowing their phosphorylation and thereby modulation of CDI.

## Introduction

Voltage-gated Ca^2+^ channels of the plasma membrane consist three subfamilies (Ca_V_1, Ca_V_2 and Ca_V_3) [1]. They are composed of 10 pore-forming α1 channel subunits and are important components of a universal cellular Ca^2+^ signaling tool kit [2]. Voltage-dependent Ca^2+^ channels are one of the main routes of cellular Ca^2+^ entry. Intracellular Ca^2+^ ions control processes as diverse as cell proliferation, neuronal development and transmitter release [2]. All of these functions have to be accomplished within a narrow range of Ca^2+^ concentrations. CDI of voltage-dependent Ca^2+^ channels is an auto-inhibitory feedback mechanism controlling Ca^2+^-influx [3], [4]. Previously we have shown that in TC neurons of the dorsal part of the lateral geniculate nucleus (dLGN), Ca^2+^-induced Ca^2+^ release (CICR) contributes to intracellular Ca^2+^ transients [5], leads to the activation of Ca^2+^-dependent K^+^ channels and thereby supports regular tonic firing [6]. Furthermore, CDI, which is under the control of multiple biochemical and activity-dependent mechanisms, has been shown to limit Ca^2+^ entry into TC neurons [7], [8], [9], [10]. Stimulation of the βAR/adenylyl cyclase (AC)/PKA-dependent pathway in TC neurons mediates behavioural state-dependent shifts in thalamic activity modes by modulating pacemaker channels, L-type Ca^2+^ channels, and Ca^2+^-dependent K^+^ channels [3], [11]. Direct application of cAMP and the catalytic subunit of PKA reduced the degree of CDI in TC neurons [9]. Although cAMP-dependent signaling and CDI represent prominent mechanisms in TC neuron function, their possible functional coupling by direct stimulation of βAR has not been investigated in these neurons yet. Recent studies in cardiac [12] as well as in hippocampal cells have shown a functional link between βAR and one type of L-type Ca^2+^ channels, namely Ca_V_1.2, via PKA [13], [14], however a possible link to CDI has not been addressed. Furthermore, AKAP has been shown to be an important element in organizing βAR-dependent pathways in neurons [13], [15], [16].

Although dephosphorylation of Ca^2+^ channels by calcineurin (PP2B) has originally been proposed to be the crucial mechanism of CDI [17], calmodulin closely tethered to the channel has been identified as the Ca^2+^ sensor and central mediator of this process [18], [19], [20]. The role of phosphorylation/dephosphorylation in CDI attracted less attention, although the close association of Ca_V_1.2 channels, phosphorylating PKA, and dephosphorylating calcineurin has been shown [17]. Based on the finding that β_2_ARs are directly associated with one of their main effector channels, namely Ca_V_1.2, via a protein complex also containing G-proteins, AC, PKA, and a counterbalancing protein phosphatase [13], it was also suggested that this protein complex might be the basis for β-adrenergic modulation of CDI [3] (Figure S1). Here we provide evidence for this hypothesis and show that stimulation of βARs reduces CDI in TC neurons via a protein complex including AKAP and PKA.

## Materials and Methods

### Ethics Statement

All experiments were carried out in accordance with the European Committees Council Directive (86/609/EEC) and approved by the local animal care committee (Landesverwaltungsamt Sachen-Anhalt, AZ: 42502/5.19 UNI MD).

### Reverse transcription-polymerase chain reaction (RT-PCR) assays

**Figure 1:**
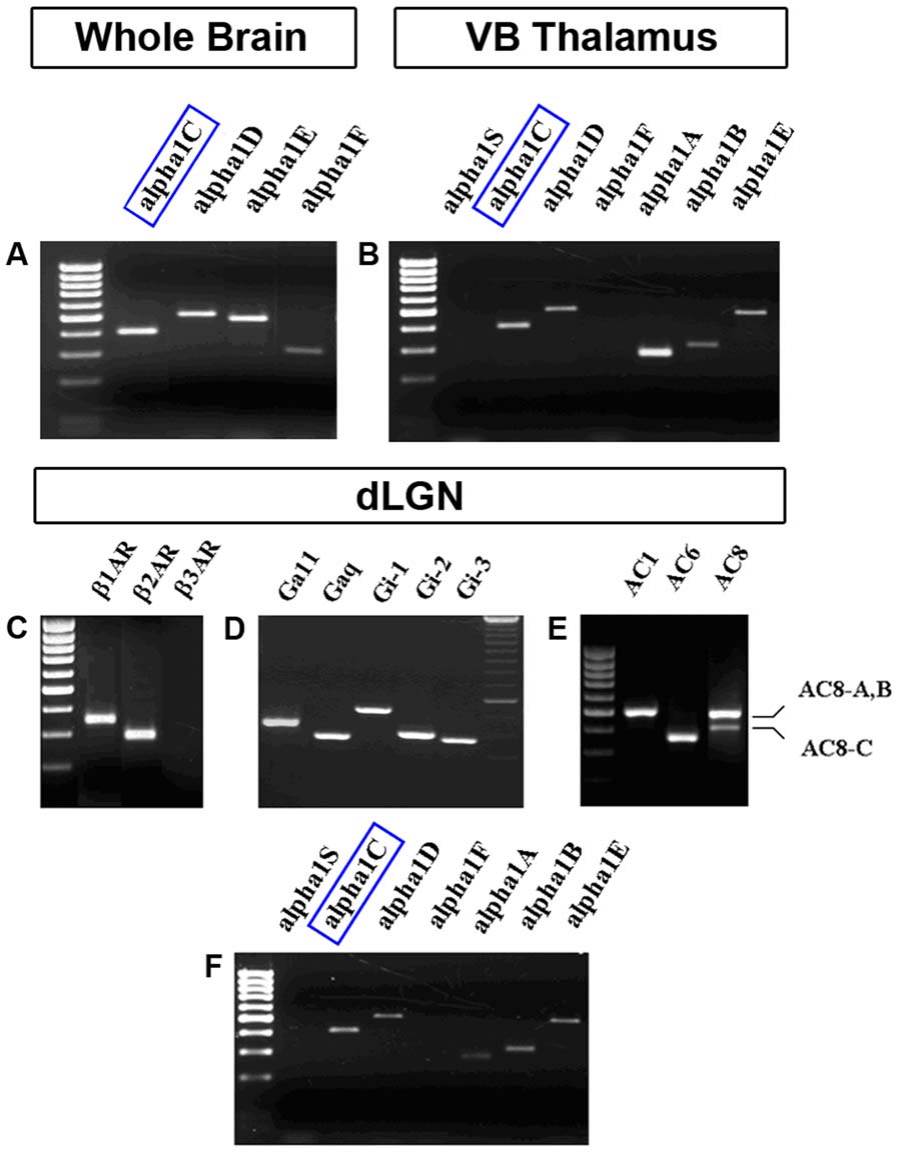
Tissue-specific expression patterns of mRNAs of the main components of βAR signaling in the dLGN of Long Evans rats. RT-PCRs of mRNA from whole brain extracts (**A**) and VB (**B**) show that the α1C subunit (colored squares) as well as other Ca^2+^ channels forms are widely expressed. The skeletal muscle form α1S was not found in both regions (as well as in dLGN, see Figure **F**). In all experiments, appropriate markers for size were used in order to assure detection of specific forms of Ca^2+^ channels. (**C**) Using specific primers for all three types of βAR, we have shown specific expression of only β_1_ and β_2_AR. β_3_AR was neither expressed in VB nor in dLGN. (**D**) Following the same procedure, we have shown the expression of some of the G proteins coupled with metabotropic receptors (Gα and Gi) in dLGN. In all experiments specific markers for size were used. (**E**) Expression of three different adenylate cyclases, namely type 1, 6 and 8 with two isoforms A/B and C in dLGN.

Poly A^+^ mRNA was prepared from freshly dissected thalamic tissue (the ventrobasal thalamic complex (VB) and dLGN were visually identified) or whole brain by extraction with Trizol reagent according to the manufacturer's instruction (Oligotex Direct mRNA, Qiagen, Hilden, Germany). First-strand cDNA synthesis was primed with oligo (dT) from 0.5–1 µg of mRNA, using the SuperScript II enzyme (Invitrogen Life Technologies). Specific primers for calcium channels α1C-α1E, G-proteins, adenylatecyclase, and β-receptors were used according to manufacturer description (Figure 1A–1F). Accession numbers for used primers were: Alpha1S (nucleotides 445–828) No. L04684; Alpha1C (nucleotides 2624–3033) No. NM012517; Alpha1D (nucleotides 3691–4200) No. NM017298; Alpha1F (nucleotides 3196–3608) No. NM0053701; Alpha1A (nucleotides 5115–5387) No. NM012918; Alpha1B (nucleotides 1742–2051) No. NM147141; Alpha1E (nucleotides 5831–6377) No. NM019294; Galpha q (nucleotides 188–560) No. AF234260; Galpha11 (nucleotides 139–554) No. AF239674; Galpha i–1 (nucleotides 669–1198) No. NM013145; Galpha i-2 (nucleotides 1125–1520) No. 031035; Galpha i-3 (nucleotides 1993–2367) No. NM013106; β1–AR (nucleotides 742–1104) No. NM012701; β2-AR (nucleotides 676–964) No. NM012492; β3-AR (nucleotides 398–797) No. NM013108; AC1 (nucleotides 1361–1863) No. XM223616; AC6 (nucleotides 1581–1940) No. NM012812; AC8 (nucleotides 3001–3480) No. NM017142; AKAP5 (nucleotides 122–589 No. NM 133515; AKAP7 (nucleotides 494–1059) No. NM 001001801; Gs (nucleotides 332–756) No. NM 019132.

### Cell type-specific RT - PCR

**Figure 2:**
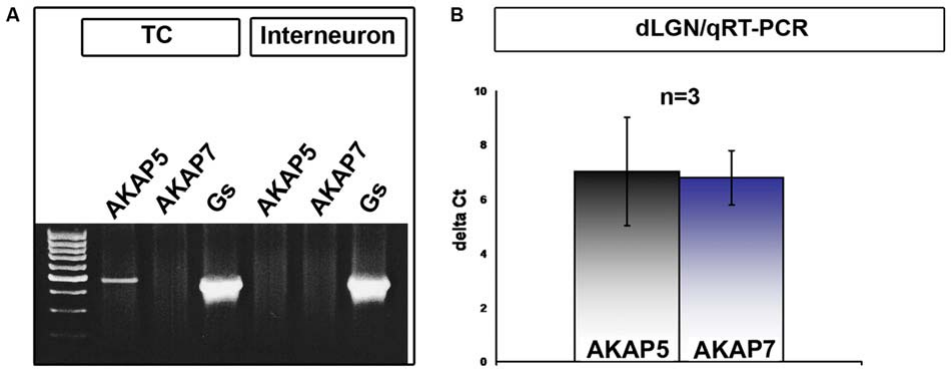
Single cell and qRT-PCR expression profiles of AKAP5, AKAP7 and the Gs subunit of G proteins. (**A**) Two different cell types are expressed in LGN, interneurons that are GAD67 positive and TC neurons that are GAD67 negative. These types of cells were collected under visual control and classified based on their morphological differences. Thereafter, mRNA was extracted. Representative PCR gel photo showing bands specific for AKAP5, AKAP7, and the Gs subunit of G proteins. Note that AKAP5 is only expressed in TC neurons while AKAP7 was not detectable. (**B**) mRNAs were isolated from the indicated brain tissues. qRT-PCR analysis showed expression of both AKAP5 and AKAP7 genes in the dLGN.

Brain tissue from P14–P24 Long Evans rats consisting of dLGN was sliced using a vibratome to 500 µM. After trypsinization of tissue blocks cells were visually identified under the microscope. Complete cells were sucked into the pipette and transferred into 3 µl carrier RNA buffer (RNeasy Micro Kit, QIAGEN) by breaking the tip of the pipette and expelling 3 µl of solution with positive pressure. The pipette solution (6 µl) was supplemented with a recombinant ribonuclease inhibitor (0.24 U/ml; RNasin; Promega, Madison, WI, USA). Cytoplasm from single, identified cells were pooled (interneurons and TC neurons, respectively), RNA was isolated without DNase treatment using an RNA isolation kit (RNeasy Micro Kit, QIAGEN), and reverse transcription (RT) protocol was used for cDNA according to manufactures protocol. Integrity of all obtained cDNAs was confirmed by using primers for the housekeeping gene GAPDH. After confirmation of integrity, cDNAs were used for standard RT-PCRs with specific primers for each gene of interest (AKAP 5; AKAP 7; AC stimulatory G-protein, Gs; Figure 2A). PCR products were separated by size on 1% agarose gels and visualised using an Eagle eye system after ethidium bromide staining.

### Quantitative *Real – time PCR*

Real-time PCR was performed using the Real PCR Master Mix 2.5X (Eppendorf) and the ABI Prism 7000 Sequence Detection System (Applied Biosystems); PCR program was: 2 min at 50°C, 10 min at 95°C, 50 cycles: 15 s at 95°C and 1 min at 60°C. Analysed probes were AKAP 5, AKAP 7, and β-2 microglobulin as the internal reference gene used for normalisation (Figure 2B). All primers were purchased from Applied Biosystems. Results were analysed with the ABI Prism 7000 SDS software. The efficiency of real-time primer/probes was nearly identical. Quantification was done using the comparative C_t_ method as described earlier [21]. In a standard PCR GAPDH (nucleotides 789–1028, accession No. NM017008) expression was checked as a positive control to confirm the integrity of transcribed cDNA. The PCR protocol for GAPDH amplification was: 3 min 94°C; 50 cycles (30 s 94°C, 1 min 61°C, 1 min 72°C); 7 min 72°C.

### Tissue Preparation

Thalamic slices were prepared from juvenile postnatal day (P) 12–P24 Long–Evans rats. After anaesthesia with isoflurane, animals were decapitated and a block of tissue containing the dLGN was rapidly removed and placed in chilled (2–4°C), oxygenated slicing solution (pH 7.35, with NaOH) containing the following (in mM): sucrose, 195; glucose, 11; Pipes, 20; KCl, 2.5; MgSO_4_, 10; and CaCl_2_, 0.5. Coronal slices of the thalamus were cut at 300 µm on a vibratome and kept submerged in artificial cerebrospinal fluid (ACSF; pH 7.35, with 95% O_2_-5% CO_2_) containing the following (in mM): NaCl, 125; KCl, 2.5; NaH_2_PO_4_, 1.25; NaHCO_3_, 22–26; MgSO_4_, 2; CaCl_2_, 2; and glucose, 10. Slices were kept for 20 min at 34°C before being cooled to room temperature, and allowed to rest for 60 to 90 min.

### Patch clamp recordings

Whole-cell recordings under voltage clamp condition were performed on visually identified TC neurons of the dLGN at room temperature (21–23°C) using borosilicate glass pipettes (GC150TF-10, Clark Electromedical Instruments, Pangbourne, UK) connected to an EPC-9/2 amplifier (HEKA Electronics, Lamprecht, Germany). Typical electrode resistances amounted to 2–4 MΩ, while access resistance was 5–15 MΩ. Series resistance compensation was routinely used (30%). Voltage-clamp experiments were governed by Pulse software (HEKA, Germany). For standard recordings the following solutions were used: (i) extracellular solution (in mM): NaCl, 125; KCl, 2.5; NaH_2_PO_4_, 1.25; NaHCO_3_, 22–26; MgSO_4_, 2; CaCl_2_, 2; and glucose, 10; TTX, 0.001; 4-AP, 4; pH 7.35 with NaOH. (ii) Intracellular solution: Cs-gluconate, 85; Cs_3_-citrate, 10; NaCl, 10; KCl, 1; EGTA. 1.1; CaCl_2_, 0.1; MgCl_2_, 0.25; HEPES, 10; TEA-Cl, 15; Mg-ATP, 3; Na_2_-GTP, 0.5; pH 7.25 with CsOH. In some experiments equimolar concentrations of ascorbic acid were added to the bath solution in order to prevent oxidation of the drugs.

**Figure 3:**
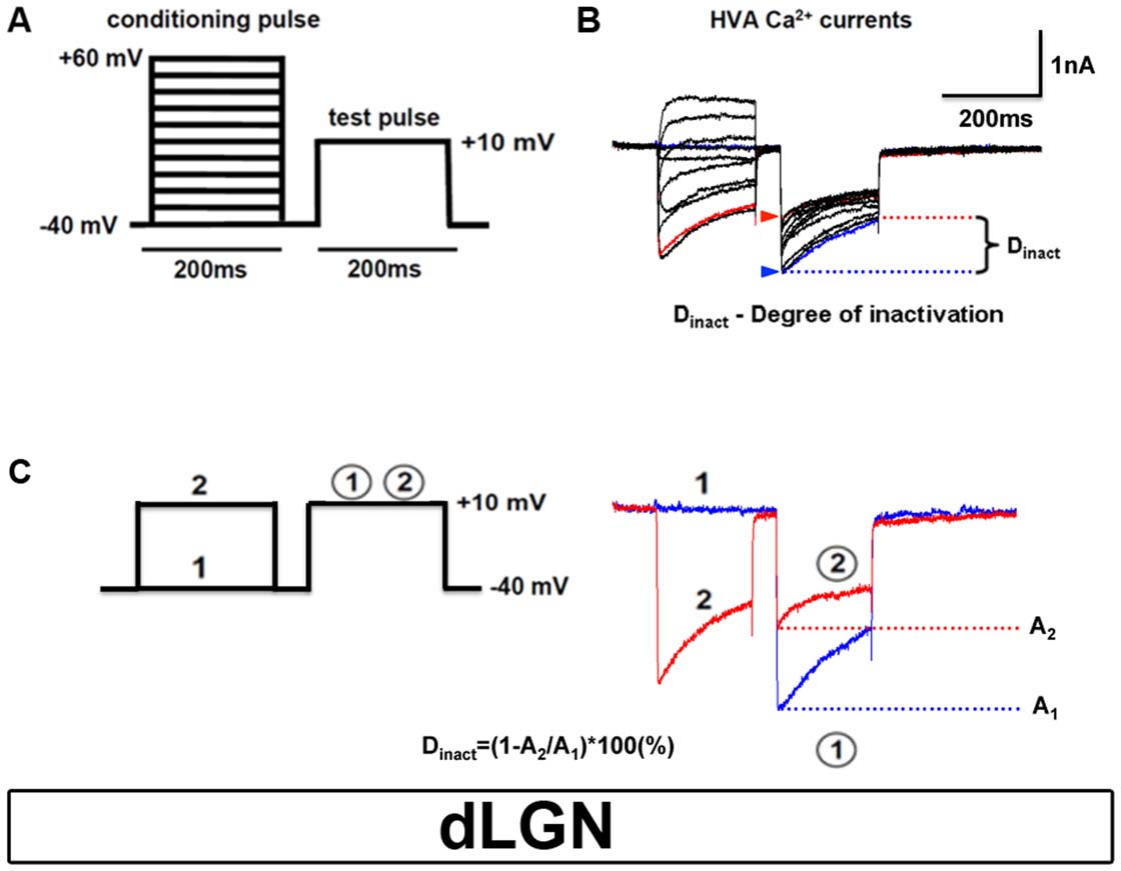
Identification of CDI in TC neurons. (**A**) Scheme of the double-pulse protocol used to elicit HVA Ca^2+^ currents. TC neurons were held at −40 mV and conditioning pulses to varying potentials (−40 to +60 mV, 200 ms duration) were followed by a brief gap (−40 mV, 50 ms) and a subsequent analyzing test pulse to a fixed potential of +10 mV (200 ms). (**B**) Family of representative current traces elicited by the pulse protocol shown in (**A**). (**C**) D_inact_ equation for calculating effects of CDI in TC neurons.

HVA Ca^2+^ currents were evoked from a holding potential of −40 mV by a double-pulse protocol in which conditioning pulses to varying potentials (−40 to +60 mV, 200 ms duration) were followed by a brief gap (−40 mV, 50 ms) and a subsequent analyzing test pulse to a fixed potential of +10 mV (200 ms) (Figure 3A). For standard recordings Ca^2+^ was used as a charge carrier and 1.1 mM EGTA was included to the intracellular solution. The degree of inactivation (D_inact_) was determined by dividing the current amplitudes elicited by a test pulse to +10 mV with maximal and minimal preceding conditioning current amplitude (Figure 3B–3C, D_inact_ = [1−(A_2_/A_1_)]×100(%)). A_2_ (I_min_, Figure 3C) represents the minimal current amplitude after a preceding conditioning pulse, as measured at the beginning of the voltage step to +10 mV (upper arrowhead in Figure 3B), and A_1_ (I_max_, Figure 3C) representing the maximal current amplitude (lower arrowhead, Figure 3B), as measured without a preceding conditioning pulse. GraphPad Prism 5.0 and Microcal Origin 6.0 software were used for data analysis and figure plotting.

### Drugs

The following drugs were used: isoproterenol hydrochloride, propranolol hydrochloride, xamoterol hemifumarate, salmeterol, BRL 37344, sodium salt, CGP 20712 dihydrochloride, ICI 118,551 hydrochloride, SR 59230A hydrochloride, okadaic acid, PKA inhibitor myristoylated PKI 14–22 amide (all from Tocris, USA). Equimolar concentrations of ascorbic acid were added to the extracellular bath solution in order to prevent oxidation of the drugs. The selective AKAP inhibitor InCELLect™ AKAP St-Ht31 and the corresponding control peptide were purchased from Promega (Germany).

### Cell culture

After incubation for 5 minutes at room temperature with 2 ml Trypsin solution, immortalized kidney cells of the African green monkey (COS-7 cells) were collected with 10 ml cell culture medium and transferred into a 15 ml Falcon tube. Cell pellets obtained after centrifugation at 500 x g for 5 minutes at 4°C were resuspended in 10 ml of Dulbecco's modified Eagle medium (DMEM; Gibco, Eggestain, Germany). Transfection was done according to the manufacturer's instructions (Invitrogen, Germany). DNA-Lipofectamine complexes were added into the flask and cells were left in incubator for the next two days. 24 hours after transfection sodium-butyrate was added in order to enhance expression of transfected proteins. 48 hours after transfection, the cells were lysed and obtained lysates were either used immediately or stored at −20°C. For GFP constructs, transfection efficiency was analyzed under a fluorescence microscope (Zeiss axioplan microscope, Carl Zeiss, Jena, Germany).

### Preparation and culturing of dissociated cell cultures from the dorsal thalamus and hippocampus

Dorsal thalami were prepared from embryos (Long-Evans rats) at stage E19 and subsequently transferred into ice-cold HBSS without Ca^2+^/Mg^2+^. After triple washing with HBSS (5 ml), 2.0 ml HBSS containing 0.5% trypsin was added to the tissue, followed by incubation at 37°C for 20 min. Thereafter tissue was washed again five times with 5 ml HBSS and finally transferred into tubes (2 ml) with HBSS containing 0.01% DNaseI. For dissociation thalamic tissue was pressed slowly three times through a 0.9 mm gauge needle followed by three passages through a 0.45 mm gauge needle. The remaining cell suspension was poured through a nylon tissue into a 50 ml tube and filled up with 18 ml DMEM. After estimating cell quantity, the suspension was diluted with DMEM in order to achieve a cell density of 30,000 cells/ml. A 500 µl aliquot of this suspension was placed on each well of a 24-well plate, containing defatted, baked, and poly-D-lysine-coated coverslips. The cell cultures were incubated at 37°C and 5% CO_2_ up to the appropriate time points. Between 3^rd^ and 4^th^ day of incubation, AraC (final concentration: 6 µM) was added to prevent further growth of glial cells.

Primary hippocampal cells were prepared in the same way as described for thalamic neurons and grown in culture until day 10 to 14. Some of the cover slips were taken out and immediately fixed with 4% paraformaldehyde (PFA), stained with PKARIIβ antibody (1∶500, BD Bioscience; see below) and used as control. For comparison cells were treated with βAR agonist isoproterenol hydrochloride (50 µM) for 5 minutes before fixation and PKARIIβ antibody staining. All cells were then incubated with the appropriate secondary antibody, mounted on microscope slides, and analysed under the microscope. Finally, distances (in pixels) between the dendritic localizations of PKA and the centre of the soma were quantified using MetaMorph software (Visitron Systems GmbH, Puchheim, Germany).

### Immunocytochemistry

After 10–14 days in vitro (DIV 10–14), PFA-fixed thalamic cells were washed two times with 25 mM glycine in phosphate buffered saline (PBS, 10 mM), one time with PBS, and subsequently preincubated at room temperature in PBS-blocking solution containing 10% horse normal serum, 2% bovine serum albumin, 5% sucrose, and 0.3% Triton X-100. After 1 h, the following primary antibodies were added in different combinations to the blocking solution and incubated for 90 min at room temperature: rabbit anti-Ca_V_1.2 (1∶200, Alomone Labs, Israel); mouse anti-cAMP-dependent protein kinase type II beta regulatory subunit (PKARIIβ, 1∶500, BD Bioscience, USA); rabbit anti-β_2_-AR (H-73 1∶400, Santa Cruz, USA); mouse anti-microtubule-associated protein 2 (MAP2, HM-2, 1∶1000, Sigma, Germany); goat anti-AKAP-150 (N-19, Santa Cruz, USA); sheep anti-protein phosphatase 2A (PP2A, Acris, Germany). Cultures were then treated with PBS including 0.3% Triton X-100, incubated with appropriate specific fluorescent secondary antibodies: Alexa 488 donkey anti-sheep IgG; Alexa 488 goat anti-mouse IgG; Alexa 488 goat anti-rabbit IgG; Alexa 488 rabbit anti-goat IgG; Alexa 647 donkey anti-mouse IgG; Alexa 647 donkey anti-goat IgG; Alexa 647 donkey anti-rabbit IgG, (all from Molecular Probes, Invitrogen, Germany); Cy3-conjugated goat anti-rabbit IgG; Cy3-conjugated goat anti-mouse IgG; Cy3-conjugated rabbit anti-goat IgG, (all from Dianova, Germany) for 90 min, washed, and mounted with Mowiol. Omission of primary and secondary antibodies resulted in lack of fluorescent signals.

### Immunohistochemistry

Long–Evans rats P25–27 were deeply anesthetized using pentobarbital (50 mg/kg body weight) and transcardially perfused with PBS, followed by an ice-cold 4% PFA/PBS for 35–40 min. Brains were removed, postfixed for 4 h in 4% PFA/PBS, and cryoprotected with 30% sucrose. Coronal sections (20 µm) were cut at the level of the dLGN, mounted onto polylysineslide glass (Menzel, Germany), and air dried. For detection of Ca_V_1.2, fresh-frozen sections were used. In this case, brains from isoflurane-anesthetized rats were removed and frozen in −50°C isopentane. Cryostat coronal sections of 20 µm thickness were cut at the level of the dLGN, thaw-mounted onto Polylysine slide glass, air dried, and fixed in 4% PFA/PBS for 10 min. After permeabilization with 0.1% Triton X-100 in PBS for 10 min and several washeswith PBS, sections were blocked with 10% normal horse serum (NHS), 2% BSA in PBS for 3 h to minimize nonspecific binding before incubation of slices with primary antibodies (rabbit anti-Ca_V_1.2, 1∶200; mouse anti-PKARIIβ, 1∶500) in 2% NHS, 2% BSA in PBS at 4°C for 16–18 h. After washing, sections were incubated withto Cy3-conjugated donkey anti- rabbit IgG (1∶400 in 2% NHS, 2% BSA in PBS, Dianova, Germany) or Alexa488 goat anti-mouse IgG (1∶750 in 2% NHS, 2% BSA in PBS, Molecular probes, Invitrogen, Germany) for 1.5 h, washed again, and mounted with Immumount.

### Culturing of COS-7 cells and pull down assays

Dulbecco's modified Eagle medium (DMEM+; Gibco, Eggestain, Germany) and washing buffer PBS were warmed to 37°C and frozen trypsin aliquots were thawed at room temperature. COS-7 cells (grown to confluency) were washed once with PBS. After incubation for 5 minutes at room temperature with trypsin (2 ml), cells were collected with 10 ml cell culture medium and transferred into a 15 ml Falcon tube. Cell pellets obtained after centrifugation at 500 x g for 5 minutes at 4°C were resuspended in 10 ml of Dulbecco's modified Eagle medium (DMEM; Gibco, Eggestain, Germany). Transfection of the cells was done according to the manufacturer's instruction (Invitrogen, Germany). Transfection was conducted for 2 days and after 24 hours sodium-butyrate was added in order to enhance expression of transfected proteins. In the following cells were lysed and lysates were used immediately or stored at −20°C. For GFP constructs, transfection efficiency was analyzed under a fluorescence microscope (Zeiss Axioplan microscope, Carl Zeiss, Jena, Germany).

Transfected COS-7 cells expressing proteins of interest tagged to GFP or c-myc were scraped in medium and transferred to a 15 ml Falcon tube. Cell flask was washed with 2 ml of Tris-buffered saline (TBS) and the solution was pulled with scraped cells. Content of the cells was made accessible by repeating two times centrifugation (1000 x g, 5 min, 4°C), discarding the supernatant, resuspension of cells in 1 ml of TBS, and transfer to a 1.5 ml Eppendorf tube. In order to disrupt cells, the pellet was frozen and thawed using liquid nitrogen. After careful resuspension in 200 µl of TBS/Triton X-100 and vortexing, the lysate was rotated for 1 h at 4°C. A cleared lysate which was obtained after 20 min centrifugation (14000 rpm, at 4°C) was diluted with TBS containing 0.2% Triton X-100 and either used directly for pull down assay or stored at −20°C for further use.

Next, 50 µl matrix beads coupled to one of the proposed binding partners with GST or MBP tag were washed 2x and equilibrated in TBS containing 0.1% Triton X-100. Appropriate lysates from COS-7 cells (200–300 µl) were added and beads were gently shaken overnight at 4°C. On the next day matrixes were centrifuged (5 min, 600 x g. 4°C) washed three times (TBS/0.2% Triton X-100, 10 min) to remove unbound proteins. Bound proteins were eluted by 5 min boiling with 4 x SDS sample buffer and either loaded on SDS-PAGE gels directly or stored at −20°C for further use.

### Immunoprecipitation

AKAP5-GFP, co-expressed with PKARIIβ-c-myc in COS-7 cells, was immunoprecipitated using µMACS™ Epitope Tag Protein Isolation Kit (Miltenyi Biotec, Germany) according to manufacturer's instructions. Eluted probes were loaded on SDS-PAGE and presence of proteins interacting with AKAP5 was detected after Western blot analysis using a PKARIIβ specific antibody (BD Bioscience, USA).

### Western blotting procedures

For immuno-blot experiments solubilized protein fractions were separated on 5–20% SDS-polyacrylamide gradient gels and subsequently transferred to nitrocellulose membranes (90 min, 200 mA). The transfer buffer contained 25 mM Tris, 192 mM glycine, 0.02% SDS and 20% methanol. After blotting, membranes were blocked with 5% dry milk and 0.1% Tween 20 in TBS for 2 h and subsequently incubated at 4°C overnight with a specific dilution of antibodies in TBSA containing 0.1% Tween 20. After final washing steps, the blots were incubated with HRP-conjugated secondary antibodies (1∶5000) for 2 h, washed again and finally developed using ECL films.

### Microscopy

Immunofluorescence analysis of cultured neurons was done using a computer-controlled inverted laser scanning microscope (Leica, Bensheim, Germany) allowing recording of Z-stacks and enabling 3D deconvolution of the obtained images. Image analysis was done using MetaMorph, ImageJ (NIH), and Adobe Photoshop CS (version 9.0 CS2) software.

### Data analysis

Statistical data analysis was done by Student's t test or one way ANOVA as indicated using GraphPad Prism 5 and Microcal Origin 6. All values were presented as mean ± SEM. As we were able to demonstrate a Gaussian distribution for the three main parameters (current amplitude, D_inact_ and ratio of inactivation) analyzed in the present study under control conditions, statistical significance was evaluated by Student's t-test. Where applicable, control values were compared with corresponding values obtained during drug application for the same cells. When intracellular substance application had to be used, an appropriate number of control cells was recorded from the same slice and used for statistical comparison. Values of P<0.05 were considered statistically significant.

## Results

### PCR expression patterns of the main components of the βAR signaling cascade in dLGN

In a first attempt to investigate the modulation of CDI via βARs, we analyzed the expression patterns of the main components of the proposed β-AR signaling pathway in dLGN TC neurons by performing RT-PCR analyses on a tissue and single cell level. Expression of RNA of the main neuronal L-type Ca^2+^ channels (Ca_V_1.2/α1C, Ca_V_1.3/α1D) were found in whole brain samples (Figure 1A), the VB (Figure 1B), and dLGN (Figure 1F). The skeletal muscle type Ca_V_1.1/α1S and the retina-specific Ca_V_1.4/α1F were not detected in thalamic tissue (Figure 1), indicating a specific RT-PCR amplification. Furthermore, the specific expression of main components of the βAR signaling cascade supposed to be involved in CDI modulation in TC neurons was confirmed in dLGN tissue (Figure 1C–1E).

The expression of AKAP and the stimulatory G protein (Gs) was probed in a cell type-specific manner using single identified cells obtained from dLGN tissue. Acutely dissociated cells were observed under an inverted microscope and small bipolar interneurons and larger multipolar TC neurons were visually identified by using established criteria [22], [23]. Single cells were collected by means of a suction pipette and used for standard PCR or quantitative real-time PCR (qRT-PCR). Because of very low amounts of the mRNA species targeted here, in some sets of experiments it was necessary to pool up to 10 cells. As Figure 2A shows, in three independent experiments (n = 3) the Gs subunit was expressed in both types of neurons, AKAP5 was only expressed in TC neurons, and AKAP7 was not detected at the single cell level. To obtain larger amounts of mRNA, we also performed qRT-PCR with samples from dLGN tissue. Using specific primers for AKAP5 and AKAP7, we were able to detect expression signals for these two genes in dLGN with almost identical expression levels (Figure 2B). Of note, that while AKAP7 is nearly equally expressed in the brain, AKAP5 exhibited a slightly higher expression level in the primary somatosensory cortex and hippocampus (data not shown). In view of positive signals for both AKAP subtypes from dLGN tissue and AKAP5 in single TC neurons, the absence of positive PCR bands for AKAP7 in extracts from single cells indicted that the amount of mRNA was below the detection limit of the method used here or pointed to an expression in other cell types present in intact tissue (glia cells, endothelial cells, blood cells).

### CDI is active in TC neurons in brain slices

Next, we demonstrated the occurrence of CDI in TC neurons in brain slices. Total HVA Ca^2+^ current, which is composed of about 40% current through L-type channels, was measured in the presence of TTX (1 µM) [24]. We have shown before that blocking of L-type calcium channels using nifedipine (1 µM), (n = 8) indeed significantly reduced CDI [5]. HVA Ca^2+^ currents were recorded in 160 cells and a double pulse voltage protocol (Figure 3A) was used to effectively disclose CDI [3]. If CDI is operative, the current evoked by the test pulse should exhibit a U-shaped dependence on the conditioning pulse potential, with maximal inactivation occurring at the peak of the conditioning pulse current-voltage (*I*–*V*) relationship. Under standard conditions the *I*–*V* relationship of the conditioning pulse demonstrated HVA Ca^2+^ currents with an activation threshold of –40 mV, maximal inward current at around +10 mV, and an apparent reversal potential at around +60 mV (data not shown). The test pulse *I*–*V* (peak amplitude of the test pulse current plotted vs. conditioning pulse voltage) revealed minimal current occurring at +10 mV (Figure 3C) as expected for a CDI mechanism. With respect to the amplitude of the test pulse current elicited from the holding potential of –40 mV, the degree of inactivation (D_inact_) was 39.5 ± 0.5% (n = 100; Figure 3C). By default, the test pulse *I*–*V* was obtained with the conditioning pulses altered in 10 mV increments from −40 mV (Figure 3A). *I*–*V* relationships were obtained as described before and all experiments were done using a protocol resulting in maximal CDI [5]. Taken together, these findings demonstrate a CDI mechanism in TC neurons in the slice preparation that is very similar to that described in these cells after acute isolation [9].

### The role of β-AR in CDI modulation of L-type Ca^2+^ channels

Since activation of the cAMP/PKA pathway is able to reduce CDI in isolated TC neurons [9], [10], activation of βARs is expected to modulate CDI of L-type calcium channels in the dLGN. βARs consist of three similar receptor subtypes (β_1_, β_2_, β_3_). Using commercially available pharmacological substances, we specifically stimulated and blocked receptor subtypes and evaluated their possible relevance for CDI modulation. First, we used general agonists and antagonists for βARs, namely isoproterenol and propranolol (data not shown). Experiments testing βAR activation alone using 10 µM isoproterenol revealed an inhibiting influence of βAR stimulation on CDI. D_inact_ was decreased to 35.7±2% (n = 4, p>0.05) as compared to control (D_inact_ = 40.1±3%). The effect of isoproterenol on CDI was reversed by co-application of the βAR antagonist propranolol (100 µM, D_inact_ = 43.7±2.1, n = 5; p>0.05; control D_inact_ = 46.1±1.6%, n = 5; data not shown). These experiments demonstrate a general role of βAR in modulation of CDI in TC neurons. The rather moderate effect of βAR on CDI may be limited by basal dephosphorylation processes in TC neurons [9] (see below).

**Figure 4:**
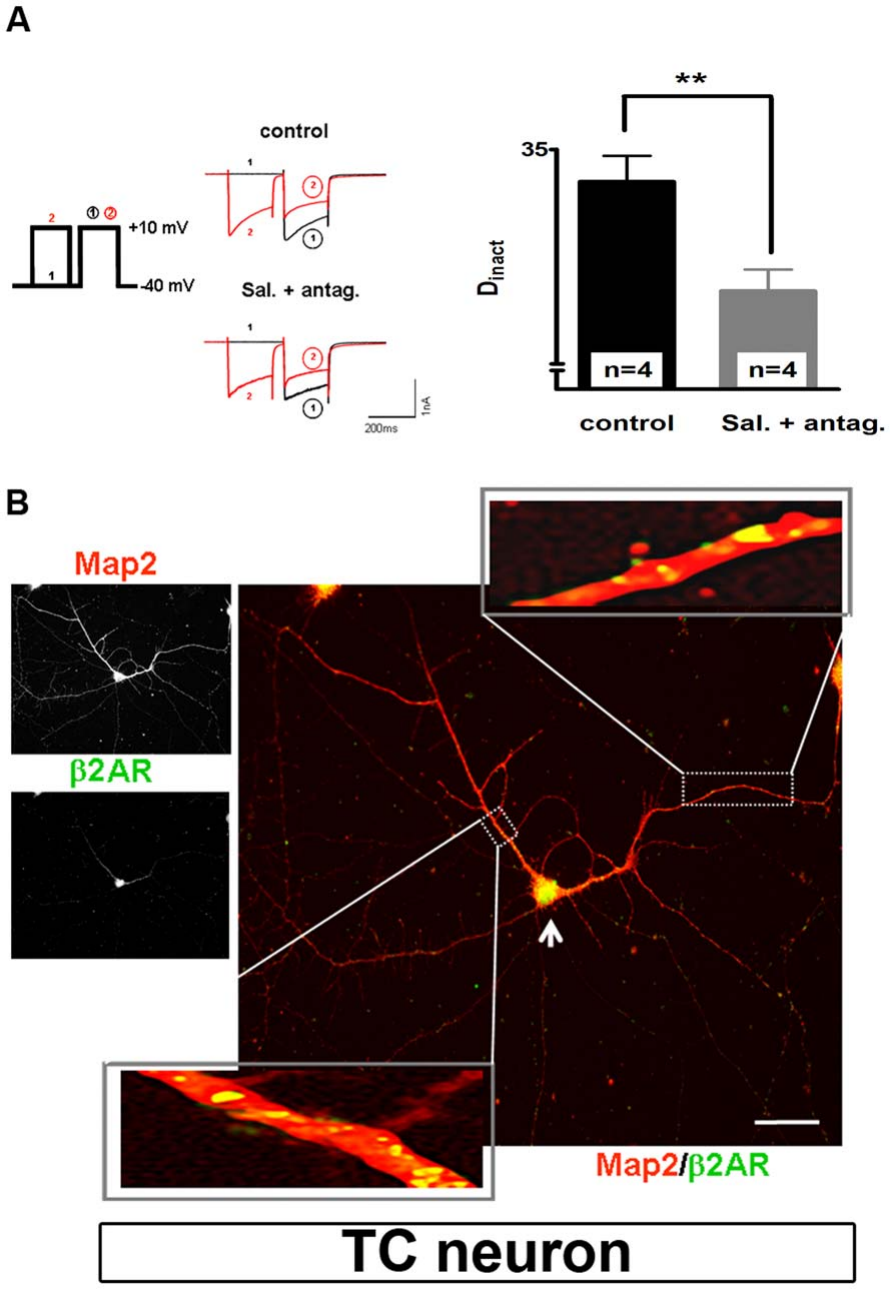
β_2_AR specifically modulates CDI in TC neurons of dLGN. (**A**) Representative current traces recorded under control conditions (upper left panel) and during extracellular application of specific β_2_AR agonist salmeterol (10 µM) plus antagonists for the other two types of receptor (CGP 20712 dihydrochloride-β_1_ antagonist and SR 59230A hydrochloride-β_3_-antagonist, 100 µM each; lower left panel) elicited by the indicated pulse protocol (middle left panel). The bar graph (right panel) represents D_inact_ under different recording conditions (as indicated). The mean value of four cells recorded under control conditions was taken for comparison with four cells recorded under 10 µM salmeterol plus 100 µM β_1_+β_3_ antagonists. Data are presented as means ± SEM of several independent experiments. **P<0.01. Significance of salmeterol plus antagonists versus control was calculated by Student's t test. The degree of inactivation is given by the normalized current amplitude of the mean postpulse I/V at +10 mV. (**B**) Immunocytochemical analysis of primary cultures of the dorsal thalamus using MAP2 specific antibody (left upper panel, red) and β_2_AR specific antibody (left down panel, green). The merged picture revealed the co-expression of these two proteins in somatic regions and proximal dendrites. Enlarged inlay represents magnification of the area indicated by the rectangle. Yellow dots represent places were these two proteins are in close proximity. Data shown are representative pictures from several independent immunostainings and preparations of neurons. In all cases, omission of primary antibodies resulted in no fluorescence signal above background (negative control).

Next, we tested agonists which bind preferentially to one of the three βAR subtypes. Therefore xamoterol (β_1_AR agonist), salmeterol (β_2_AR agonist), and BRL 37344, (β_3_AR agonist) were used. Challenging TC neurons with xamoterol (10 µM), reduced the degree of inactivation from 43.8±2.3% to 37.4±1.8% (n = 4, p<0.05). This decrease was similar to that induced by salmeterol (10 µM) application (reduction of D_inact_ from 39.0±2.2% to 34.5±0.3%, n = 4, p<0.05; data not shown) and BRL 37344 (10 µM) application (reduction of D_inact_ from 43.8±0.6% to 38.3±0.8%, n = 4, p<0.05; data not shown). To minimize side effects of each drug on non-preferred βAR subtypes and to further enhance the specificity of our pharmacological approach, we used agonists that prefers one type of receptor (in 10 µM concentration) in combination with antagonists for the other two receptor types (in 100 µM concentration), thereby allowing to more selectively investigate the role of each type of receptor in CDI modulation. The following antagonists were applied: CGP 20712 (β_1_AR antagonist), ICI 118,551 (β_2_AR antagonist), and SR 59230A (β_3_AR antagonist). Using this approach, effects on CDI were detected only when the β_2_AR agonist salmeterol was used in combination with CGP 20712 and SR 59230A (reduction of D_inact_ from 33.5±1.2% to 28.4±1%, n = 4; Figure 4A). For the other two βAR subtypes, no significant modulation of CDI was obtained in this set of experiments (data not shown). These results clearly demonstrate that β_2_AR specifically contribute to CDI modulation in dLGN TC neurons.

### Localization of β_2_ARs in cultured TC neurons

Next, we analyzed the specific expression and localization of β_2_AR in 10 days old cultured thalamic neurons and performed double immunostaining using an antibody against β_2_AR (Figure 4B) in combination with an antibody against the dendritic marker protein microtubule associated protein 2 (Map2; Figure 4B). The expression of both proteins was detected with β_2_AR mainly localized in somatic and proximal dendrites of TC neurons (Figure 4B, merge image), cellular compartments that are involved in modulation of CDI in TC neurons.

### Blocking of PKA suppresses βAR-dependent modulation of CDI in TC neurons

**Figure 5:**
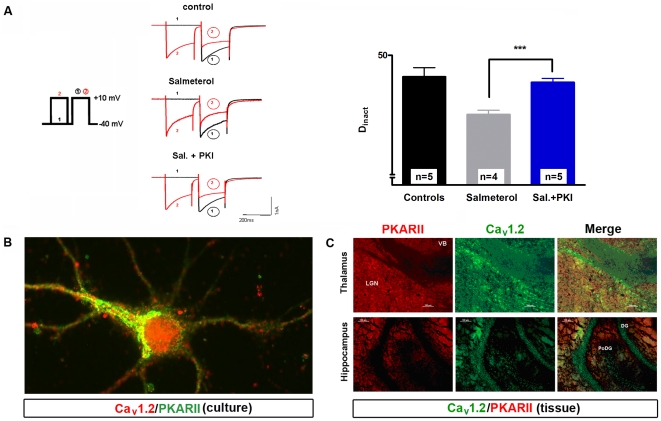
Inhibition of PKA completely suppresses the effect of βAR stimulation on CDI in TC neurons. (**A**) Representative current traces recorded under control conditions (upper left panel) and in the presence of the specific β_2_AR agonist salmeterol alone (10 µM; left middle panel) or in combination with PKI 14–22 amide (10 µM; lower left panel). The bar graph represents D_inact_ under different recording conditions (as indicated). The mean value of five cells recorded under control conditions was taken for comparison with four cells recorded under 10 µM salmeterol and five cells recorded under 10 µM salmeterol plus PKI 14–22 amide. Data are presented as means ± SEM of several independent experiments. ***P<0.001. Significance of salmeterol plus PKI (n = 5) versus salmeterol alone (n = 4) was calculated by Student's t test. The degree of inactivation is given by the normalized current amplitude of the mean postpulse I/V at +10 mV. (**B**) Close co-expression of the main modulator of CDI, PKA (green) and Ca_V_1.2 (red) in cultured neurons. Yellow dots represent places were these two proteins are in close proximity. Data shown are representative pictures from several independent immunostainings and preparations of neurons. In all cases, omission of primary antibodies resulted without signal (negative control). (**C**) Indicated brain regions were immunostained with antibodies specific for PKARIIβ and Ca_V_1.2. Thalamic regions LGN and VB revealed very strong interaction patterns in merged pictures. Association of these proteins is still present in hippocampus but on lower level. DG (dentate gyrus), PoDG (polymorph layer of the dentate gyrus).

Next, we assessed the possible contribution of PKA in CDI modulation of TC neurons by intracellular application of a PKA inhibitor (PKI, 10 µM) in whole cell patch clamp experiments. As shown in Figure 5A, the degree of inactivation was significantly reduced from 44.6±2.3% (n = 5) under control conditions to 35.1±1.1% (n = 4, p<0.001; Figure 5A) when TC neurons were challenged with salmeterol (10 µM), while in combination with PKI the inhibitory effect of βAR stimulation was absent (D_inact_ = 43.2±1%; n = 5). These findings indicate that the modulation of CDI in TC neurons depends on the activity of PKA.

To address the possible association between PKA and Ca_V_1.2, we performed double immunostainings of cultured TC neurons treated with the general βAR agonist isoproterenol. In this set of experiments we used an antibody directed against the regulatory subunit IIβ of PKA (PKARIIβ), which is highly expressed in thalamus and hippocampus (Figure 5C). Image analysis pointed to a close spatial proximity of the two proteins at the somato-dendritic junction following βAR stimulation (Figure 5B). On the other hand untreated cells showed more somatic expression of the PKARIIβ (data not shown).

### AKAPs play a crucial role in the modulation of CDI in TC neurons

**Figure 6:**
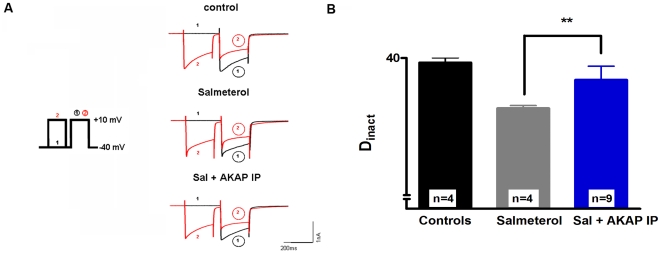
Inhibition of PKA binding to AKAPs suppresses the effect of βAR stimulation in TC neurons. (**A**) Representative current traces recorded under control conditions (upper left panel) and in the presence of the specific β_2_AR agonist salmeterol alone (10 µM; left middle panel) or in combination with InCELLect™ AKAP St-Ht31 inhibitory peptide (10 µM; lower left panel). (**B**) The bar graph represents D_inact_ under different recording conditions (as indicated). The mean value of four cells recorded under control conditions was taken for comparison with four cells recorded under 10 µM salmeterol and nine cells recorded under 10 µM salmeterol plus InCELLect™ AKAP St-Ht31 inhibitory peptide. Data are presented as means ± SEM of several independent experiments. **P<0.01. Significance of salmeterol (n = 4) versus salmeterol plus InCELLect™ AKAP St-Ht31 inhibitory peptide (n = 9) was calculated by Student's t test. The degree of inactivation is given by the normalized current amplitude of the mean postpulse I/V at +10 mV.

Next, we investigated the possible contribution of AKAP in CDI modulation in TC neurons. AKAP is assumed to simultaneously bind to PKA, Ca^2+^ channels, and protein phosphatases, including calcineurin [17]. Therefore a selective AKAP inhibitory peptide which binds to the PKA binding sites of AKAP thereby blocking the binding of intracellular PKA and a corresponding control peptide which allows normal PKA binding were used. When salmeterol (10 µM) was applied in the presence of intracellular AKAP inhibitory peptide (10 µM), the degree of inactivation (37.6±1.5%, n = 9; Figure 6A and 6B) was comparable to control conditions (39.5±0.5, n = 4, p>0.05) but different from conditions where salmeterol was applied alone (34.5±0.3%, n = 4, p<0.01; Figure 6A and 6B). Using the same experimental protocol, application of salmeterol in the presence of intracellular control peptide did not change the reduction in CDI (data not shown). In summary, these experiments point to a role of AKAP in the modulation of CDI in TC neurons.

### Complex formation by the main components of the β-AR signaling cascade in TC neurons

**Figure 7:**
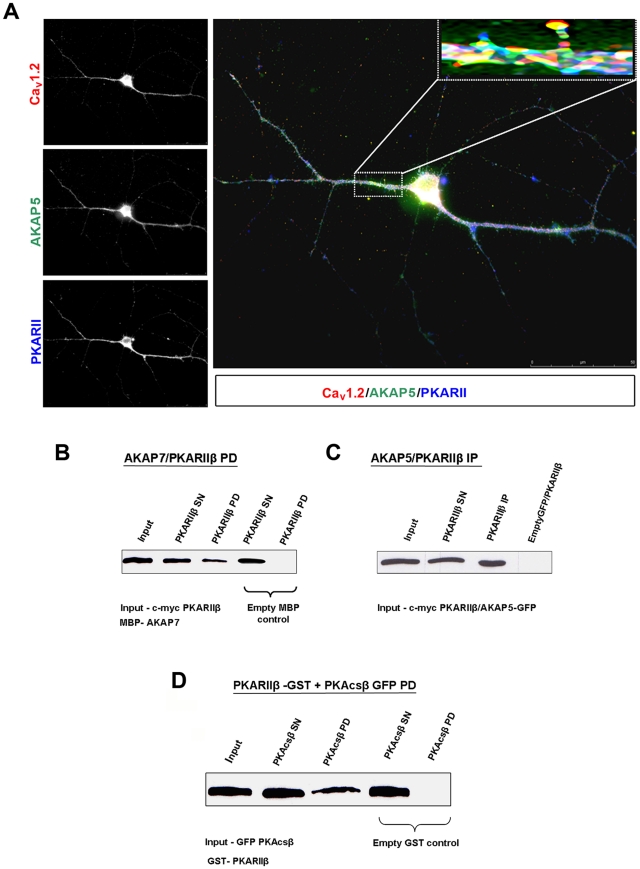
Interaction partners important for CDI modulation in TC neurons. (**A**) Immunocytochemical analysis of primary cultures of the dorsal thalamus using Ca_V_1.2- (red), AKAP150- (green) and PKARIIβ- (blue) specific antibodies. Merged picture shows the close connection of the components of the proposed ternary complex, especially in somatic regions and proximal dendrites. Enlarged inlay represents a magnification of the area indicated by the rectangle. Data shown are representative pictures from several independent immunostainings and preparations of neurons. In all cases, omission of primary antibodies resulted without signal (negative control). Western blot analysis and pull down assays were done as described in "Methods". (**B**) Interaction of AKAP7-MBP and PKARIIβ-c-myc was detected using antibodies against c-myc. (**C**) IP of PKARIIβ-c-myc and AKAP5-GFP detected after incubation with GFP-coupled magnetic beads using antibodies derived against PKARIIβ. (**D**) Existence of PKA holoenzyme consisting of PKARIIβ-GST and PKAcsβ-GFP was detected with antibodies against GFP protein.

After stimulation of the β-AR signaling cascade, it is assumed that a ternary complex is formed between PKA, AKAP, and Ca_V_1.2, the formation of which is important for CDI modulation in TC neurons. In order to find evidence corroborating this assumption, we next performed immunocytochemical staining using antibodies specific for Ca_V_1.2 (Figure 7A, upper left panel), AKAP 5 (Figure 7A, middle left panel), and PKARIIβ (Figure 7A, lower left panel), in 10 days old cultured thalamic neurons. Fluorescence imaging revealed that all three proteins are densely expressed in somatic regions and proximal dendrites of TC neurons. Merged images pointed to rather close spatial association of the three proteins (Figure 7A, right panel). In contrary to Ca_V_1.2, another calcium channel Ca_V_2.1 is similarly expressed at somatic regions and proximal dendrites but also additionally in distal dendrites (Figure S2).

### Protein-protein interactions between components of the ternary inactivation complex

Since immunocytochemical stainings pointed to the possibility that PKA, AKAP, and Ca_V_1.2 are located close to each other, thereby contributing to the CDI process, we performed pull down assays which confirmed an interaction between PKARIIβ and AKAP7 (Figure 7B) as well as PKARIIβ and PKAcsβ (Figure 7D). Control incubations of samples of interest with the appropriate fusion-protein partner (empty vector) showed no signal in Western blots. Moreover, indications for an interaction of PKARIIβ with another member of the AKAP family, namely AKAP5, were obtained by co-immunoprecipitation experiments. Therefore, magnetic beads coupled to GFP antibody and Western blotting using a PKARIIβ-specific antibody were used to verify the binding of PKARIIβ to GFP-tagged AKAP5 (Figure 7C). Control incubations of samples of interest (c-myc PKARIIβ) with the appropriate fusion-protein partner (empty GFP vector) showed no signal in Western blots. These results provided further evidence for a close coupling between PKA and AKAP in the thalamus.

### Phosphorylation and dephosphorylation processes in modulation of CDI

**Figure 8:**
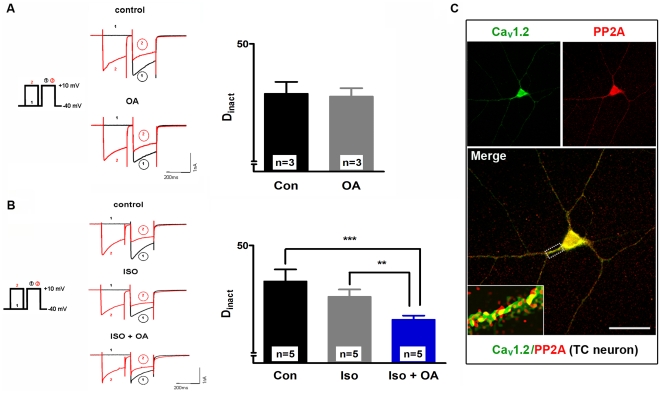
The role of dephosphorylation processes in CDI modulation of TC neurons. (**A**) Representative current traces recorded under control conditions (upper left panel) and in the presence of okadaic acid (OA) (10 µM; left down panel). The bar graph (right panel) represents D_inact_ under different recording conditions (as indicated). The mean value of three cells recorded under control conditions was taken for comparison with three cells recorded under 10 µM OA. (**B**) Representative current traces recorded under control conditions (upper left panel) and in the presence of the isoproterenol alone (10 µM; left middle panel) or in combination with OA (10 µM; lower left panel). The bar graph (right panel) represents D_inact_ under different recording conditions (as indicated). The mean value of five cells recorded under control conditions was taken for comparison with five cells recorded under 10 µM isoproterenol and five cells recorded under isoproterenol plus OA. Data are presented as means ± SEM of several independent experiments. ***P<0.001. Significance of isoproterenol plus OA (n = 5) versus controls (n = 5) was calculated by Student's t test. **P<0.01. Significance of isoproterenol (n = 5) versus isoproterenol plus OA (n = 5) was calculated by Student's t test. The degree of inactivation is given by the normalized current amplitude of the mean postpulse I/V at +10 mV. (**C**) Immunocytochemical analysis of primary cultures of the dorsal thalamus using Ca_V_1.2- (left panel, green) and PP2A-specific antibodies (right panel, red). Merge picture showed close association of the two proteins, especially in somatic regions and proximal dendrites. Data shown are representative pictures from several independent immunostainings and thalamic neurons preparations. In all cases, omission of primary antibodies resulted in no fluorescence signal above background (negative control).

It is well documented that phosphorylation/dephosphorylation processes play an important role in the regulation of calcium channel activity [3], [9]. In an additional series of patch clamp experiments we focused on the role of dephosphorylation in the modulation of CDI in TC neurons after βAR stimulation. The double-pulse protocol was applied using the phosphatase inhibitor okadaic acid (OA; 10 µM alone; p>0.05; Figure 8A), or in combination with isoproterenol (10 µM; Figure 8B). After blocking dephosphorylation processes, the degree of inactivation is reduced to 29.3±1.1% (n = 5) in comparison to βAR stimulation alone (35.7±2%, n = 5; p <0.01), and non-stimulated control cells which showed D_inact_ of 40±3.3% (n = 5; p <0.001) in these experiments.

Next, the expression of protein phosphatases PP2A was investigated in 10 days old cultured thalamic neurons by using antibodies for PP2A (Figure 8C, upper right panel) and Ca_V_1.2 (Figure 8C, upper left panel). Both proteins were mainly localized in somatic regions and proximal dendrites of TC neurons (Figure 8C, lower panel). Merged pictures pointed to a close spatial arrangement.

## Discussion

The present study provides clear evidence that the CDI of HVA Ca^2+^channels is decreased by βAR signaling in central neurons. Moreover, we present findings that are in agreement with the existence of a possible protein complex including PKA, AKAP, and Ca_V_1.2 which underlies the modulation of CDI. The most important findings of the present study are: (i) Classical double pulse protocols reveal the occurrence of CDI in TC neurons in a slice preparation; (ii) Activation of β_2_AR induces the down-regulation of CDI; (iii) Blocking of PKA signaling completely suppresses the effect of βAR stimulation on CDI, (iv) AKAP might play a crucial role in CDI modulation and the docking of PKA to sites close to Ca_V_1.2 (see Text S1). Moreover, blocking of the interaction between AKAP and PKA significantly reduces CDI and translocation of PKA. (v) Phosphorylation and dephosphorylation processes represent the basis for bidirectional up- and down-regulation of CDI in TC neurons, respectively.

### βAR stimulation and modulation of CDI of Ca_V_1.2 via phosphorylation and dephosphorylation processes

We have previously demonstrated the existence of CDI of HVA Ca^2+^ currents in TC neurons in different (acutelyisolated cells, brain slices) thalamic preparations [5], [8], [9], [10]. Based on these findings, the basic features of CDI in TC neurons can be defined as follows: (i) Under control conditions the degree of inactivation varies between about 35–40%. (ii) In addition to L-type Ca^2+^ channels, Q-type channels are also governed by CDI. (iii) CDI is influenced by a number of cellular mechanisms including repetitive neuronal activity, phosphorylation and dephosphorylation, Ca^2+^-binding proteins, the cytoskeleton, and intracellular Ca^2+^ release. The present study adds to these findings by demonstrating the specific influence of β_2_AR stimulation via PKA and scaffolding proteins of the AKAP family on the degree of CDI.

In the present study, we demonstrated that stimulation of βAR, which leads to phosphorylation of Ca^2+^ channels in a cAMP-dependent manner via PKA activation, significantly reduced the degree of inactivation of L-type Ca^2+^ channels. The same results were obtained when channel dephosphorylation was inhibited. This indicates that phosphorylation keeps L-type Ca^2+^ channels in a state of high open probability, ready to open during depolarization [9], [25]. The modulation of L-type Ca^2+^ channels through phosphorylation via different second messenger systems is well established, and includes phosphorylation by PKA and CaM kinase II (for review, see [26], [27]). Both types of modulation result in an increase in peak current amplitude [28], [29]. Furthermore, CDI has been shown to be reduced by activation of cAMP-dependent phosphorylation [9], [25], [30]. Therefore, both the increment in HVA Ca^2+^ current amplitude and the reduction of CDI after βAR stimulation observed in the present study are consistent with a phosphorylation of L-type Ca^2+^ channels by PKA.

The serine residue at position 1928 (Ser1928) of Ca_V_1.2 channels is one important target of PKA activity in heart and brain [31], [32], [33]. However, it has been recently shown that mutation of Ser1928 of cardiac L-type Ca^2+^ channels has only a small effect on channel phosphorylation after βAR stimulation [34], pointing to the existence of additional phosphorylation sites [34], [35], [36]. Moreover, meaningful regulation of channel activity by PKA phosphorylation requires a proper balance with dephosphorylation processes. Several studies in hippocampal neurons have shown that protein phosphatases including PP1, PP2A, and calcineurin directly bind to the C-terminal region of Ca_V_1.2 [17], [31], [33]. Moreover, the signaling pathway from β_2_AR to the Ca_V_1.2 channel, including G-proteins, adenylate cyclase, PKA and the counterbalancing protein phosphatases, forms a closely associated protein complex in the forebrain [13]. However, the role of this complex in context of CDI modulation has not been addressed yet. Ca_V_1.2 channels are expressed in TC neurons [8] and the existence of βARs, coupled positively to adenylate cyclase in these neurons, has been shown [37]. Moreover, we previously demonstrated effects of calyculin A and ascomycin, which are blockers of PP1, PP2A, and calcineurin [38] on CDI and Ca^2+^ current amplitude in TC neurons [9]. The present study on brain slices confirmed and extended previous findings obtained in acutely isolated TC neurons by identifying β_2_AR as the receptor subtype involved in CDI modulation and showing that blocking protein phosphatases by okadaic acid, has a significant effect on CDI during β-AR stimulation. From our data, it is therefore reasonable to conclude that PKA and protein phosphatases antagonistically modulate CDI of Ca_V_1.2 channels in TC neurons via phosphorylation and dephosphorylation processes.

Although our experiments with okadaic acid demonstrated the role of dephosphorylation processes in modulation of CDI after βAR stimulation in TC neurons, the specific type of phosphatase involved in TC neurons is still not clear. The original model of CDI in *Helix aspersa* included a dephosphorylation cycle by calcineurin as the fundamental step leading to channel closure [39]. Later, evidence for and against an involvement of calcineurin was found [17], [40], [41], [42], [43], [44], [45]. In TC neurons, application of the calcineurin blocker ascomycin boosts CDI [9], [46]. Although these observations clearly indicate a modulation of CDI by calcineurin in TC neurons, the inactivation process itself is probably not a dephosphorylation reaction. As shown above, PKA is the main enzyme which phosphorylates Ca_V_1.2 channels, thereby keeping them in an open state (increasing their open probability) and restraining the effects of CDI. Besides this phosphorylation processes that occur after βAR stimulation, there might be a constant dephosphorylation driven by PP1 and PP2.

### AKAP mediates the modulation of Ca_V_1.2 channel during βAR stimulation

Ca_V_1.2 channels can physically associate with either AKAP5 or AKAP7 through a leucine zipper interaction [17], [47]. Both, the modulation of channels and its downstream signaling depend upon the identity of the associated AKAPs. Although both AKAP subtypes target PKA to the channel, AKAP5 also targets calcineurin and thereby confers unique characteristics upon AKAP5-complexed L-type channels in neurons [17]. AKAP5 is the major AKAP protein in neurons, where it is widely distributed and has been shown to anchor protein kinases and other signaling proteins to multiple receptors and ion channels [48]. On the one side, AKAP5 recruits PKA and calcineurin to the AMPA receptor [49], associates with Ca_V_1.2 [14], [50] in neurons, recruits PKA and calcineurin to the channel and is necessary for the βAR stimulation of L-type calcium currents [13], [14], [48] as well as for the L-type calcium current-mediated activation of the transcriptional regulator NFATc4 [17]. On the otherside, AKAP5 binds also to β_2_AR and facilitates receptor phosphorylation and signaling [51]. Moreover, colocalization with Ca_V_1.2 and postsynaptic density (PSD) proteins in dendritic spines of hippocampal neurons has been shown [52]. Three different binding sites for AKAP5 were described in the N terminus, the cytoplasmic loop connecting repeats I and II, and in the C terminus of Ca_V_1.2 [14]. The C-terminal leucine zipper was shown to be essential for AKAP binding and for βAR stimulation and reversible phosphorylation of Ca_V_1.2 in heart muscle and in neurons [17], [47]. Mutation of the three basic residues of this motif blocked AKAP5 and PKA binding and phosphorylation of the Ca^2+^ channel in response to βAR stimulation [17]. Moreover, mutation of known binding sites on Ca_V_1.2 for the scaffold proteins, like AKAP and PSD proteins did not change membrane expression of Ca_V_1.2 [52]. The above findings indicate that binding of these proteins is necessary for the regulation of Ca_V_1.2 after βAR stimulation but does not have an influence on the membrane localization of Ca_V_1.2.

Based on the previous findings discussed above and the results presented here, we propose the following role for AKAP in the regulation of CDI in TC neurons. Following βAR stimulation, AC gets activated via G-proteins and produces cAMP which then activates PKA. With support of AKAPs, PKA targets its final effector, the Ca_V_1.2 channels and phosphorylates them (see Figure S1). In the present study we demonstrated that blocking the binding of AKAP to PKA significantly reduces CDI. Moreover, under similar conditions we have shown that PKA phosphorylation of Ca_V_1.2 and translocation of this enzyme close to the channel depends on binding of PKA and AKAPs in hippocampal neurons (see Text S1 & Figure S3). Most recent studies demonstrated that AKAP5 is required to localize both RIIa and RIIb containing holoenzymes to the dendritic regions of neurons in the hippocampus and striatum. PKA is dramatically delocalized within dendrites in both the KO and D36 (mutant that lacks the PKA binding domain of AKAP5) mice indicating that no other AKAP subtype is able to compensate and maintain normal PKA localization [53]. Our study confirmed an interaction between AKAP and PKA in pull down assays and demonstrated that after stimulation of the βAR signaling cascade a ternary complex is formed between PKA, AKAP, and Ca_V_1.2 and that the formation of this complex is important for CDI modulation in TC neurons. Moreover, as mentioned before, AKAP5 also targets calcineurin which is able to activate protein phosphatase PP1 [54] and therefore might have multiple function in regulation of CDI by influencing both phosphorylation by PKA and dephosphorylation processes.

### Functional significance of Ca^2+^ channel phosphorylation after β-AR stimulation

Release of transmitters from a number of brainstem terminals modulates the behavioural states of an individual by depolarizing TC relay neurons [37]. During states of slow-wave sleep, thalamic relay neurons are hyperpolarized and display rhythmic burst activity [11]. During states of wakefulness, these cells are depolarized and display tonic single spike activity, resulting in the faithful transmission of sensory signals through the dorsal thalamus. The shift from burst activity to tonic activity is mediated by increased activity of ascending brainstem fibres that are thought to increasingly release acetylcholine (ACh), noradrenaline (NA) and serotonin (5-HT) during wakefulness. Both NA via β-receptors and 5-HT via an unknown 5-HT receptor subtype, activate adenylate cyclase [37] in TC relay neurons and are thus able to positively modulate HVA Ca^2+^ currents.

When attempting to integrate the findings of the present study into the known framework of thalamic physiology it can be assumed that HVA Ca^2+^ currents are especially activated during tonic firing. Furthermore, following release of NA, HVA Ca^2+^ current amplitudes will be increased while CDI is decreased. Another consequence of βAR stimulation may be the AC/cAMP-dependent inhibition of high conductance Ca^2+^-dependent K^+^ (BK_Ca_) channels in sensory TC neurons [55]. In addition, tonic sequences of action potentials are coupled to CICR from intracellular stores, thereby further increasing Ca^2+^ entry into TC neurons during wakefulness [6]. It has been shown that intracellular Ca^2+^ release provides Ca^2+^ that contributes to CDI and activates BK_Ca_ channels in TC neurons [5], [6]. Computer modelling indicated that activation of BK_Ca_ channels leads to the occurrence of spike frequency adaptation (P. Meuth & T. Budde, unpublished observations), a condition that would impair the faithful 1∶1 relay of incoming trains of sensory action potentials. These data indicate a fine-tuned interplay between activity dependent Ca^2+^ influx, phosphorylation/dephosphorylation processes and the mode of activity, possibly to enable faithful signal integration and transfer during wakefulness.

Future studies will have to unravel the different modulatory pathways that act upstream of the multiple CDI mechanisms thereby pointing to additional functions of CDI and unraveling further the elusive role of HVA Ca^2+^ channels in thalamic physiology.

## Supporting Information

Text S1 **AKAPs assist in PKA translocation from somatic regions to the plasma membrane. **Translocation of PKARIIβ after βAR stimulation with isoproterenol in hippocampal neurons.
(DOC)

Figure S1 **Main components of the βAR signaling cascade in dLGN.**
(TIF)

Figure S2 **Differential expression of Ca_V_2.1 from Ca_V_1.2 in TC neurons of thalamus.** Immunocytochemical analysis of primary cultures of the dorsal thalamus using Ca_V_2.1- (green) and MAP2-specific (neuronal marker, red) antibodies. Merge image showed expression of these two proteins in somatic, proximal and in distal regions of TC neurons. Data shown are representative pictures from several independent experiments. In all cases, omission of primary antibodies resulted in staining without signal (negative control).
(TIF)

Figure S3 **PKA translocation from somatic regions to the plasma membrane.** (**A**) Cultured hippocampal cells were treated with 50 µM isoproterenol or kept under control conditions (control/no treatment), fixed and immunostained with PKARIIβ antibody. Enlarged inlays represent magnification of the area indicated by the rectangle. Note translocation of PKA from somatic perinuclear region in control cells to more distal regions close to the plasma membrane and probably Ca^2+^ channels in cells treated with isoproterenol. (**B**) Ten days old hippocampal cultures were treated for 5 minutes with 50 µM isoproterenol in combination with 50 µM control peptide AKAP St-Ht31 (left panel), 50 µM isoproterenol in combination with 50 µM AKAP St-Ht31 inhibitory peptide (right panel) and 50 µM isoproterenol in combination with 7.5 µM Anisomycine (down panel). The cells were then fixed and immunostained with PKARIIβ antibody. Note that PKA is still translocated to proximal dendrites, after blocking the association of PKA with AKAPs, translocation is almost completely inhibited and anisomycine treatment did not block PKA translocation. (**C**) Quantification of translocation experiments using MetaMorph was done by measurement of PKA distance from centre of the soma to dendrites in pixels after different treatments. Data are presented as means ± SEM of several independent experiments. ***P<0.001, Anova test.
(TIF)

## References

[1] Lacinova L (2005). Voltage-dependent calcium channels.. Gen Physiol Biophys.

[2] Berridge MJ, Lipp P, Bootman MD (2000). The versatility and universality of calcium signalling.. Nat Rev Mol Cell Biol.

[3] Budde T, Meuth S, Pape HC (2002). Calcium-dependent inactivation of neuronal calcium channels.. Nat Rev Neurosci.

[4] Bardo S, Cavazzini MG, Emptage N (2006). The role of the endoplasmic reticulum Ca2+ store in the plasticity of central neurons.. Trends Pharmacol Sci.

[5] Rankovic V, Ehling P, Coulon P, Landgraf P, Kreutz MR (2010). Intracellular Ca2+ release-dependent inactivation of Ca2+ currents in thalamocortical relay neurons.. Eur J Neurosci.

[6] Budde T, Sieg F, Braunewell KH, Gundelfinger ED, Pape HC (2000). Ca2+-induced Ca2+ release supports the relay mode of activity in thalamocortical cells.. Neuron.

[7] Armstrong DL (1989). Calcium channel regulation by calcineurin, a Ca2+-activated phosphatase in mammalian brain.. Trends Neurosci.

[8] Meuth S, Budde T, Pape H-C (2001). Differential control of high-voltage activated Ca2+ current components by a Ca2+-dependent inactivation mechanism in thalamic relay neurons.. Thalamus & Related Systems.

[9] Meuth S, Pape HC, Budde T (2002). Modulation of Ca2+ currents in rat thalamocortical relay neurons by activity and phosphorylation.. Eur J Neurosci.

[10] Meuth SG, Kanyshkova T, Landgraf P, Pape HC, Budde T (2005). Influence of Ca2+-binding proteins and the cytoskeleton on Ca2+-dependent inactivation of high-voltage activated Ca2+ currents in thalamocortical relay neurons.. Pflugers Arch.

[11] Steriade M, Jones EG, McCormick D (1997). Thalamus & Related Systems.

[12] Balijepalli RC, Foell JD, Hall DD, Hell JW, Kamp TJ (2006). Localization of cardiac L-type Ca(2+) channels to a caveolar macromolecular signaling complex is required for beta(2)-adrenergic regulation.. Proc Natl Acad Sci U S A.

[13] Davare MA, Avdonin V, Hall DD, Peden EM, Burette A (2001). A beta2 adrenergic receptor signaling complex assembled with the Ca2+ channel Cav1.2.. Science.

[14] Hall DD, Davare MA, Shi M, Allen ML, Weisenhaus M (2007). Critical role of cAMP-dependent protein kinase anchoring to the L-type calcium channel Cav1.2 via A-kinase anchor protein 150 in neurons.. Biochemistry.

[15] Davare MA, Dong F, Rubin CS, Hell JW (1999). The A-kinase anchor protein MAP2B and cAMP-dependent protein kinase are associated with class C L-type calcium channels in neurons.. J Biol Chem.

[16] Marshall MR, Clark3rd JP, Westenbroek R, Yu FH, Scheuer T (2011). Functional Roles of a C-terminal Signaling Complex of CaV1 Channels and A-kinase Anchoring Protein 15 in Brain Neurons.. J Biol Chem.

[17] Oliveria SF, Dell'Acqua ML, Sather WA (2007). AKAP79/150 anchoring of calcineurin controls neuronal L-type Ca2+ channel activity and nuclear signaling.. Neuron.

[18] Pitt GS, Zuhlke RD, Hudmon A, Schulman H, Reuter H (2001). Molecular basis of calmodulin tethering and Ca2+-dependent inactivation of L-type Ca2+ channels.. J Biol Chem.

[19] Soldatov NM (2003). Ca2+ channel moving tail: link between Ca2+-induced inactivation and Ca2+ signal transduction.. Trends Pharmacol Sci.

[20] Cens T, Rousset M, Leyris JP, Fesquet P, Charnet P (2006). Voltage- and calcium-dependent inactivation in high voltage-gated Ca(2+) channels.. Prog Biophys Mol Biol.

[21] Budde T, Caputi L, Kanyshkova T, Staak R, Abrahamczik C (2005). Impaired regulation of thalamic pacemaker channels through an imbalance of subunit expression in absence epilepsy.. J Neurosci.

[22] Pape HC, Budde T, Mager R, Kisvarday ZF (1994). Prevention of Ca(2+)-mediated action potentials in GABAergic local circuit neurones of rat thalamus by a transient K+ current.. J Physiol.

[23] Broicher T, Kanyshkova T, Landgraf P, Rankovic V, Meuth P (2007). Specific expression of low-voltage-activated calcium channel isoforms and splice variants in thalamic local circuit interneurons.. Mol Cell Neurosci.

[24] Budde T, Munsch T, Pape HC (1998). Distribution of L-type calcium channels in rat thalamic neurones.. Eur J Neurosci.

[25] You Y, Pelzer DJ, Pelzer S (1995). Trypsin and forskolin decrease the sensitivity of L-type calcium current to inhibition by cytoplasmic free calcium in guinea pig heart muscle cells.. Biophys J.

[26] Catterall WA (1997). Modulation of sodium and calcium channels by protein phosphorylation and G proteins.. Adv Second Messenger Phosphoprotein Res.

[27] Rossie S (1999). Regulation of voltage-sensitive sodium and calcium channels by phosphorylation.. Adv Second Messenger Phosphoprotein Res.

[28] Sculptoreanu A, Rotman E, Takahashi M, Scheuer T, Catterall WA (1993). Voltage-dependent potentiation of the activity of cardiac L-type calcium channel alpha 1 subunits due to phosphorylation by cAMP-dependent protein kinase.. Proc Natl Acad Sci U S A.

[29] Xiao RP, Cheng H, Lederer WJ, Suzuki T, Lakatta EG (1994). Dual regulation of Ca2+/calmodulin-dependent kinase II activity by membrane voltage and by calcium influx.. Proc Natl Acad Sci U S A.

[30] Kalman D, O'Lague PH, Erxleben C, Armstrong DL (1988). Calcium-dependent inactivation of the dihydropyridine-sensitive calcium channels in GH3 cells.. J Gen Physiol.

[31] Davare MA, Horne MC, Hell JW (2000). Protein phosphatase 2A is associated with class C L-type calcium channels (Cav1.2) and antagonizes channel phosphorylation by cAMP-dependent protein kinase.. J Biol Chem.

[32] Hulme JT, Westenbroek RE, Scheuer T, Catterall WA (2006). Phosphorylation of serine 1928 in the distal C-terminal domain of cardiac CaV1.2 channels during beta1-adrenergic regulation.. Proc Natl Acad Sci U S A.

[33] Hall DD, Feekes JA, Arachchige Don AS, Shi M, Hamid J (2006). Binding of protein phosphatase 2A to the L-type calcium channel Cav1.2 next to Ser1928, its main PKA site, is critical for Ser1928 dephosphorylation.. Biochemistry.

[34] Lemke T, Welling A, Christel CJ, Blaich A, Bernhard D (2008). Unchanged beta-adrenergic stimulation of cardiac L-type calcium channels in Ca v 1.2 phosphorylation site S1928A mutant mice.. J Biol Chem.

[35] Ganesan AN, Maack C, Johns DC, Sidor A, O'Rourke B (2006). Beta-adrenergic stimulation of L-type Ca2+ channels in cardiac myocytes requires the distal carboxyl terminus of alpha1C but not serine 1928.. Circ Res.

[36] Xu H, Ginsburg KS, Hall DD, Zimmermann M, Stein IS (2010). Targeting of protein phosphatases PP2A and PP2B to the C-terminus of the L-type calcium channel Ca v1.2.. Biochemistry.

[37] McCormick DA (1992). Neurotransmitter actions in the thalamus and cerebral cortex and their role in neuromodulation of thalamocortical activity.. Prog Neurobiol.

[38] Herzig S, Neumann J (2000). Effects of serine/threonine protein phosphatases on ion channels in excitable membranes.. Physiol Rev.

[39] Chad JE, Eckert R (1986). An enzymatic mechanism for calcium current inactivation in dialysed Helix neurones.. J Physiol.

[40] Branchaw JL, Banks MI, Jackson MB (1997). Ca2+- and voltage-dependent inactivation of Ca2+ channels in nerve terminals of the neurohypophysis.. J Neurosci.

[41] Schuhmann K, Romanin C, Baumgartner W, Groschner K (1997). Intracellular Ca2+ inhibits smooth muscle L-type Ca2+ channels by activation of protein phosphatase type 2B and by direct interaction with the channel.. J Gen Physiol.

[42] Victor RG, Rusnak F, Sikkink R, Marban E, O'Rourke B (1997). Mechanism of Ca(2+)-dependent inactivation of L-type Ca2+ channels in GH3 cells: direct evidence against dephosphorylation by calcineurin.. J Membr Biol.

[43] Lukyanetz EA, Piper TP, Sihra TS (1998). Calcineurin involvement in the regulation of high-threshold Ca2+ channels in NG108-15 (rodent neuroblastoma x glioma hybrid) cells.. J Physiol.

[44] Burley JR, Sihra TS (2000). A modulatory role for protein phosphatase 2B (calcineurin) in the regulation of Ca2+ entry.. Eur J Neurosci.

[45] Zeilhofer HU, Blank NM, Neuhuber WL, Swandulla D (2000). Calcium-dependent inactivation of neuronal calcium channel currents is independent of calcineurin.. Neuroscience.

[46] Kawai M, Lane BC, Hsieh GC, Mollison KW, Carter GW (1993). Structure-activity profiles of macrolactam immunosuppressant FK-506 analogues.. FEBS Lett.

[47] Hulme JT, Lin TW, Westenbroek RE, Scheuer T, Catterall WA (2003). Beta-adrenergic regulation requires direct anchoring of PKA to cardiac CaV1.2 channels via a leucine zipper interaction with A kinase-anchoring protein 15.. Proc Natl Acad Sci U S A.

[48] Bauman AL, Goehring AS, Scott JD (2004). Orchestration of synaptic plasticity through AKAP signaling complexes.. Neuropharmacology.

[49] Colledge M, Dean RA, Scott GK, Langeberg LK, Huganir RL (2000). Targeting of PKA to glutamate receptors through a MAGUK-AKAP complex.. Neuron.

[50] Dai S, Hall DD, Hell JW (2009). Supramolecular assemblies and localized regulation of voltage-gated ion channels.. Physiol Rev.

[51] Fraser ID, Cong M, Kim J, Rollins EN, Daaka Y (2000). Assembly of an A kinase-anchoring protein-beta(2)-adrenergic receptor complex facilitates receptor phosphorylation and signaling.. Curr Biol.

[52] Di Biase V, Obermair GJ, Szabo Z, Altier C, Sanguesa J (2008). Stable membrane expression of postsynaptic CaV1.2 calcium channel clusters is independent of interactions with AKAP79/150 and PDZ proteins.. J Neurosci.

[53] Weisenhaus M, Allen ML, Yang L, Lu Y, Nichols CB (2010). Mutations in AKAP5 disrupt dendritic signaling complexes and lead to electrophysiological and behavioral phenotypes in mice.. PLoS One.

[54] Guerini D (1997). Calcineurin: not just a simple protein phosphatase.. Biochem Biophys Res Commun.

[55] Biella G, Meis S, Pape -CH (2001). Modulation of a Ca2+-dependent K+-current by intracellular cAMP in rat thalamocortical relay neurons.. Thalamus & Related Systems.

