# Supplementary material for: Modulation of Calcium-Dependent Inactivation of L-Type Ca2+ Channels via β-Adrenergic Signaling in Thalamocortical Relay Neurons
Source: PLoS One. 2011 Dec 2;6(12):e27474. doi: 10.1371/journal.pone.0027474 (PMC3229489; doi:10.1371/journal.pone.0027474)
Supplement: Text S1 — AKAPs assist in PKA translocation from somatic regions to the plasma membrane. Translocation of PKARIIβ after βAR stimulation with isoproterenol in hippocampal neurons. (DOC) [file pone.0027474.s001.doc]

**Text S1**

**AKAPs assist in PKA translocation from somatic regions to the plasma membrane**

We explored the possibility that PKA translocates close to its target channels after βAR stimulation. In brain slices, both hippocampal as well as thalamic cells strongly express Ca_V_1.2 and PKARIIβ (Figure 5C). Since cultured hippocampal neurons proved to be more robust than thalamic cells and represent a widely used model system to investigate neuronal function, hippocampal neurons at 10 days in culture were used in the following. From PKARIIβ staining it was evident that in control cells PKARIIβ was mostly localised in somatic perinuclear regions (Figure S3A, left panel), whereas after isoproterenol treatment PKA translocated from this regions to more distal sites and most probably close to the membrane and Ca^2+^ channels (Figure S3B, right panel). Note that translocated PKA shows a specific staining pattern in dendrites (looking like straight lines) giving us a reason to believe that this movement is supported by scaffolding proteins, namely the cytoskeleton (microtubule) and probably proteins from the AKAP family.

Thus, it was reasonable to test a possible role of AKAP in PKA translocation after βAR stimulation. According to our hypothesis, PKA cannot be translocated to Ca^2+^ channels when the binding of intracellular PKA to AKAPs is blocked. Application of AKAP inhibitory peptide strongly decreased PKA translocation induced by isoproterenol (50 µM) treatment (Figure S3B, upper right panel), while the control peptide had no significant effect (Figure S3B, upper left panel). The use of anisomycine (7.5 µM), a blocker of *de novo* protein synthesis, showed that PKA translocation is due to the stimulation of functional βAR in the plasma membrane and is not a consequence of synthesis of new receptors (Figure S3B, lower panel). Significant differences were found between the group of cells treated with isoproterenol alone and the group treated with isoproterenol together with AKAP inhibitory peptide but not control peptide (Figure S3C).
