# Supplementary figures and images for: Modulation of Calcium-Dependent Inactivation of L-Type Ca2+ Channels via β-Adrenergic Signaling in Thalamocortical Relay Neurons
Source: PLoS One. 2011 Dec 2;6(12):e27474. doi: 10.1371/journal.pone.0027474 (PMC3229489; doi:10.1371/journal.pone.0027474)

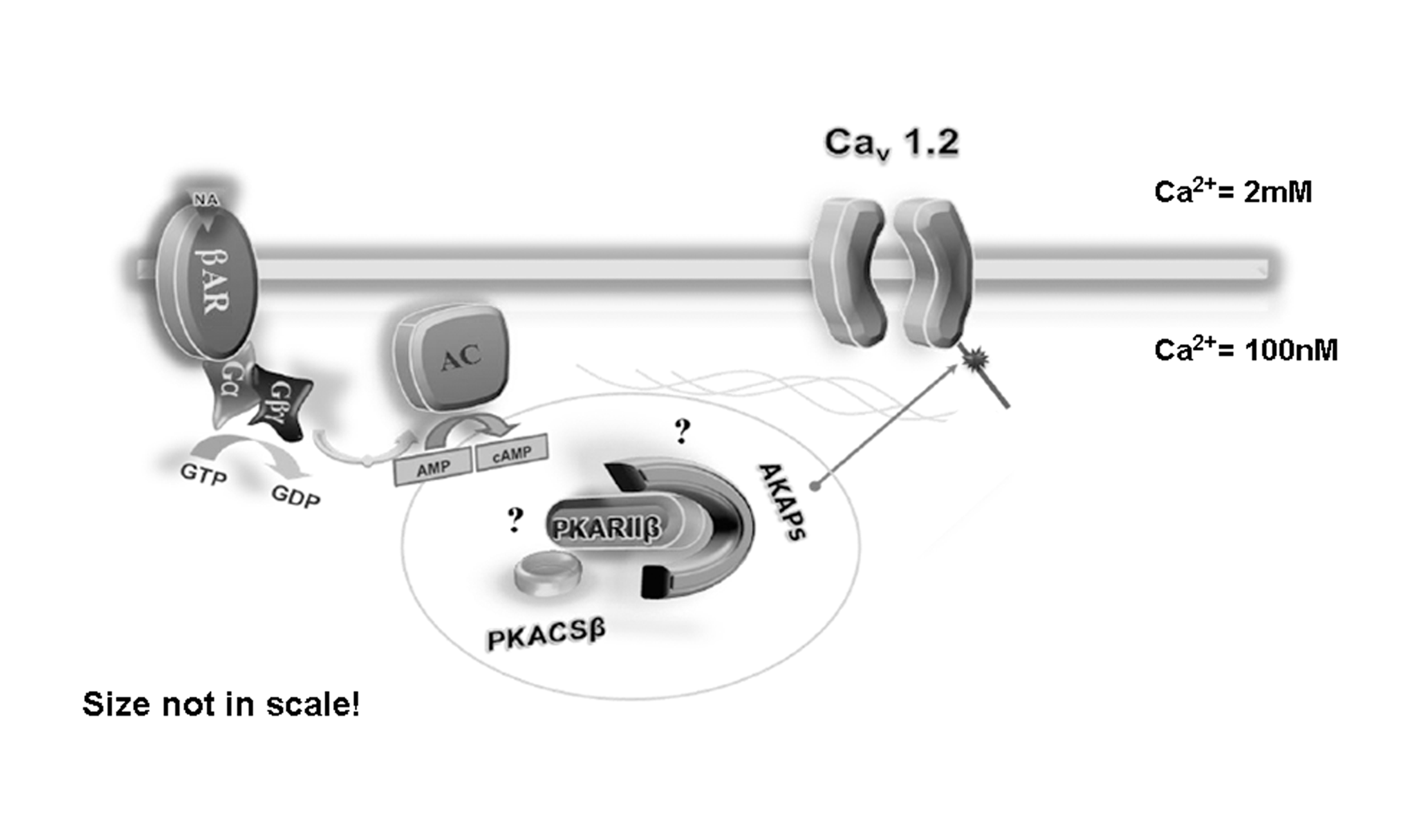

Supplement: Figure S1 — Main components of the βAR signaling cascade in dLGN. (TIF) [file pone.0027474.s002.tif]

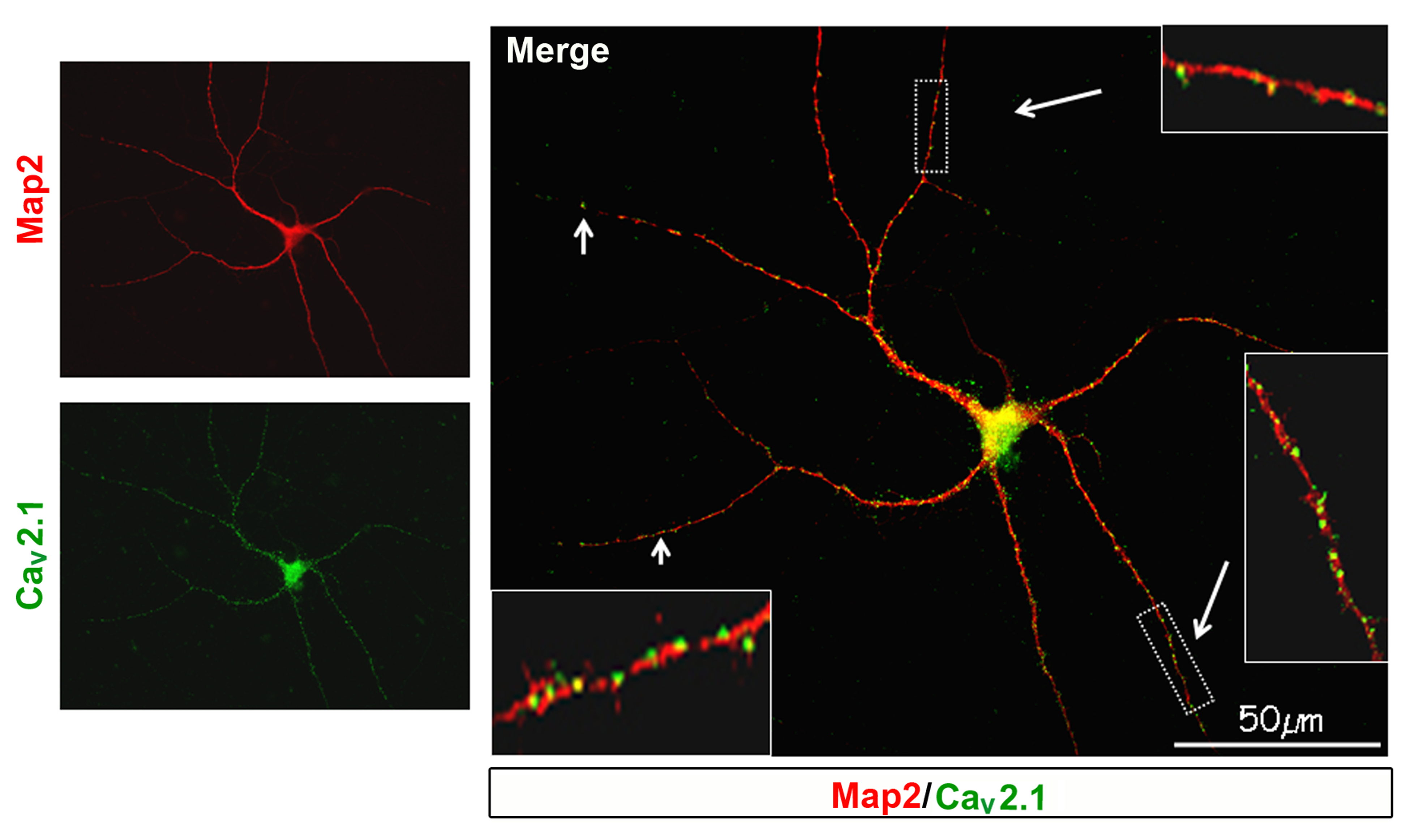

Supplement: Figure S2 — Differential expression of CaV2.1 from CaV1.2 in TC neurons of thalamus. Immunocytochemical analysis of primary cultures of the dorsal thalamus using CaV2.1- (green) and MAP2-specific (neuronal marker, red) antibodies. Merge image showed expression of these two proteins in somatic, proximal and in distal regions of TC neurons. Data shown are representative pictures from several independent experiments. In all cases, omission of primary antibodies resulted in staining without signal (negative control). (TIF) [file pone.0027474.s003.tif]

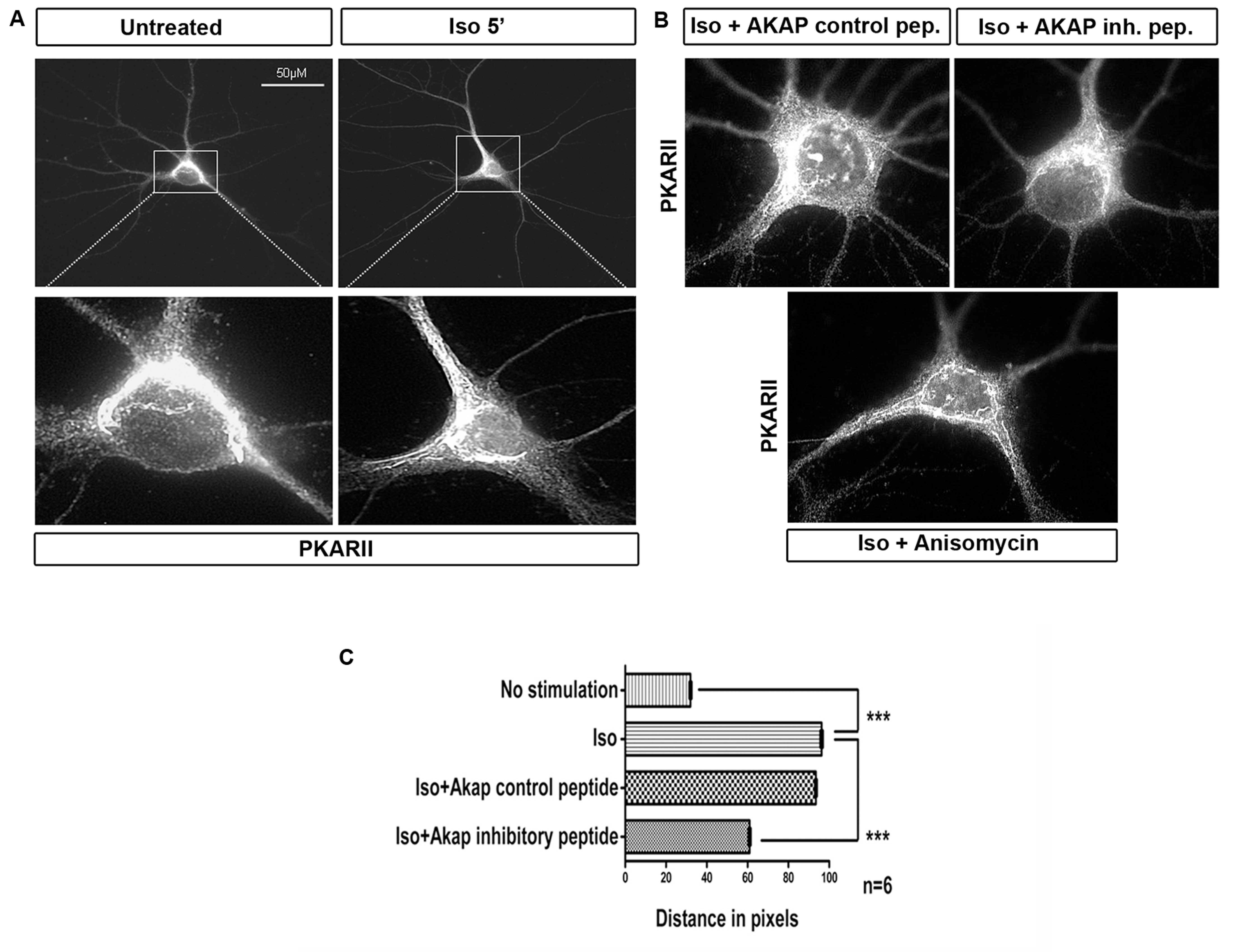

Supplement: Figure S3 — PKA translocation from somatic regions to the plasma membrane. (A) Cultured hippocampal cells were treated with 50 µM isoproterenol or kept under control conditions (control/no treatment), fixed and immunostained with PKARIIβ antibody. Enlarged inlays represent magnification of the area indicated by the rectangle. Note translocation of PKA from somatic perinuclear region in control cells to more distal regions close to the plasma membrane and probably Ca2+ channels in cells treated with isoproterenol. (B) Ten days old hippocampal cultures were treated for 5 minutes with 50 µM isoproterenol in combination with 50 µM control peptide AKAP St-Ht31 (left panel), 50 µM isoproterenol in combination with 50 µM AKAP St-Ht31 inhibitory peptide (right panel) and 50 µM isoproterenol in combination with 7.5 µM Anisomycine (down panel). The cells were then fixed and immunostained with PKARIIβ antibody. Note that PKA is still translocated to proximal dendrites, after blocking the association of PKA with AKAPs, translocation is almost completely inhibited and anisomycine treatment did not block PKA translocation. (C) Quantification of translocation experiments using MetaMorph was done by measurement of PKA distance from centre of the soma to dendrites in pixels after different treatments. Data are presented as means ± SEM of several independent experiments. ***P<0.001, Anova test. (TIF) [file pone.0027474.s004.tif]
